# Unraveling Shade Tolerance and Plasticity of Semi-Evergreen Oaks: Insights From Maritime Forest Live Oak Restoration

**DOI:** 10.3389/fpls.2019.01526

**Published:** 2019-11-20

**Authors:** Emily C. Thyroff, Owen T. Burney, Michael V. Mickelbart, Douglass F. Jacobs

**Affiliations:** ^1^Department of Forestry and Natural Resources, Hardwood Tree Improvement and Regeneration Center, Purdue University, West Lafayette, IN, United States; ^2^John T. Harrington Forestry Research Center, New Mexico State University, Las Cruces, NM, United States; ^3^Department of Botany and Plant Pathology, Purdue University, West Lafayette, IN, United States

**Keywords:** Quercus virginiana, canopy openness, ecophysiology, gas exchange, leaf traits, light acclimation, plant competition, forest regeneration

## Abstract

*Quercus* spp. (oaks) are generally intermediate in shade tolerance, yet there is large variation within the genus in shade tolerance and plasticity in response to varying resource availability. Ecophysiological knowledge specific to semi-evergreen *Quercus* spp. from subtropical maritime forests is lacking relative to temperate deciduous oaks. We studied the influence of light availability and plant competition on leaf physiology and performance of semi-evergreen *Quercus virginiana* on a barrier island along the US southern Atlantic coast. Seedlings were underplanted in pine (*Pinus taeda*) plantation stands with varying overstory density (clear-cut, heavy thin, light thin, and non-thinned; creating a gradient of understory light availability) and vegetation (no competition removal or herbaceous competition removal) treatments. After 2 years, seedling survival was higher with increasing light availability (clear-cut = heavy thin > light thin > non-thinned). Seedling growth (i.e., diameter, height, and crown width) increased similarly with increasing thinning intensity, while vegetation control was mainly beneficial to seedling growth in clear-cuts. These responses were partially explained by foliar nitrogen and leaf trait measurements, which followed the same pattern. *Q. virginiana* seedlings demonstrated high plasticity in their ability to acclimate to varying resource availability, as indicated by light response curves, specific leaf area, stomatal density, stomatal pore index, and maximum theoretical stomatal conductance. Light compensation and saturation points illustrated seedling capacity to increase net CO_2_ assimilation with increased light availability. Leaves on trees in the high light environment had the highest net CO_2_ assimilation, stomatal density, stomatal pore index, maximum theoretical stomatal conductance, and lowest specific leaf area. Although we demonstrated the relative shade tolerance of *Q. virginiana* in lower light environments (i.e., heavy and light thin plots), this semi-evergreen species shows high plasticity in capacity to respond to varying resource availability, similar to other *Quercus* spp. from mesic and Mediterranean environments.

## Introduction

Plasticity is an adaptive strategy to promote survival and fitness of long-lived forest tree species that may experience several environmental changes and associated stresses throughout their lifespan (Cavender-Bares and Ramírez-Valiente, 2017; Gil-Pelegrín et al., 2017a). Phenotypic plasticity, both physiological and morphological, affects life history traits and contributes to the large distribution ranges (Gratani et al., 2003; Niinemets, 2015) and wide ecophysiological variation (Niinemets and Valladres, 2006; Gil-Pelegrín et al., 2017b) of *Quercus* spp. (oaks). From deciduous to semi-evergreen or evergreen *Quercu*s spp., plasticity has commonly been identified as a trait contributing to greater drought, cold, and shade tolerance (Valladares et al., 2002; Gimeno et al., 2009; Limousin et al., 2012; Ramírez-Valiente et al., 2017). Plasticity relates to how populations may respond to climate change as well as the development of improved management activities (Paquette et al., 2007; Gimeno et al., 2009). Plasticity is also important in understanding mechanisms that lead to regeneration and restoration success (Benito-Garzón et al., 2013; Lawson and Michler, 2014; Löf et al., 2019).

*Quercus* spp. are generally intermediate in shade tolerance and underplanting may provide an effective means to restore these species (Dey et al., 2012). Increased light and soil moisture resulting from overstory density reduction may benefit planted *Quercus* seedlings, but also pioneer species (Dey et al., 2008; Dey et al., 2012; Kern et al., 2012; Villar-Salvador, 2016). Pioneer species that acclimate rapidly and take advantage of higher light levels are particularly competitive, often suppressing oak seedling survival and growth (Paquette et al., 2006; Dey et al., 2008; Gardiner et al., 2010). Limited light, nutrients, and water resources from competition can negatively affect physiological processes, inhibiting seedling performance (Salifu et al., 2009; Grossnickle, 2012). Removal of competing vegetation, therefore, has potential to channel limited resources to planted seedlings, yet can be logistically prohibitive (Wagner and Zasada 1991; Fleming et al., 2006). Alternatively, maintaining partial overstory may introduce sufficient light to optimize growth in the target species, while restricting faster growing competition (Elliott and Swank, 1994; Paquette et al., 2006; Brown et al., 2014).

To facilitate forest restoration, there has been increased investigation in converting pine plantations to diverse hardwood forests using clear-cutting or thinning followed by planting of desired species (Parker et al., 2001; Gómez-Aparicio et al., 2009; Löf et al., 2010; Villar-Salvador, 2016; Lesko and Jacobs, 2018). Overstory removal treatments affect light, temperature, soil moisture, and soil compaction in complex feedback loops, which are dependent on species and ecosystems (Canham et al., 1990; Madrigal-González et al., 2017; Soto et al., 2017). Thus, understanding how silvicultural treatments affect plasticity and adaptive potential of target species regeneration may accelerate restoration processes by increasing availability of light, water, and nutrients.

Maritime forests of the subtropical US southern Atlantic coast, characterized by the dominant, semi-evergreen *Quercus virginiana* L., represent a case study where conversion of pine plantations back to diverse native hardwood forests may facilitate the restoration of associated ecosystem services (Albers and Alber, 2003; Jones et al., 2013). Within the range of *Q. virginiana*, there has been centuries of human land transformation, particularly on the more stable land where maritime forests develop (Bratton and Miller, 1994; Bellis, 1995; Fox et al., 2007; Jones et al., 2013). A fraction of the original estimated land area of maritime forests remains at approximately 39,000 ha, which has created interest to protect and restore maritime forests (Mathews et al., 1980; Lopazanski et al., 1988).

Many agricultural lands that were originally maritime forests on the US southern Atlantic coast were abandoned and more recently planted into pine plantations (i.e., *Pinus taeda* L.) for commercial investment and to minimize erosion (Fox et al., 2007; Brockerhoff et al., 2008). Pine plantations tend to perform poorly when exposed to inherent coastal stressors and abandoned monoculture pine plantations with low genetic diversity are particularly prone to disease and outbreaks of southern pine beetles (*Dendroctonus frontalis* Zimm.), which are an economically destructive forest pest due to exponential outbreaks (Conner et al., 2005; Fox et al., 2007; Brockerhoff et al., 2008; Watson et al., 2013; Nowak et al., 2015; Asaro et al., 2017). Clear-cuts are used to salvage residual timber value and reduce continual spread of active outbreak sites, while overstory thinning helps to minimize future outbreaks in at-risk stands (Belanger et al., 1993; Watson et al., 2013; Asaro et al., 2017). The complete or partial removal of the pine overstory provides an opportunity to restore maritime forest by regenerating *Q. virginiana*.

Our objective was to better understand the underlying physiological mechanisms that drive plant structure, physiology, and function of *Q. virginiana*, as a semi-evergreen oak. *Quercus virginiana* L. has a broad distribution across maritime edaphic site factors compared to other maritime oaks (Cavender-Bares and Pahlich 2009), suggesting potential for high plasticity. While the effects of varying resources such as temperature and precipitation has been studied in *Q virginiana* (Kurtz et al., 2013; Ramírez-Valiente et al., 2015; Cavender-Bares and Ramírez-Valiente, 2017), the species response to light and competition has yet to be explored. We experimentally evaluated the relative influence of pine overstory density and vegetation control treatments on *Q. virginiana* seedling performance. We hypothesized that *Q. virginiana* survival, growth, and leaf development would peak in the thinned treatments, when competition was controlled, reflective of the relative shade tolerance of most *Quercus* spp. Under this scenario, seedlings should show higher net CO_2_ assimilation and greater growth and development due to increased light compared to the control, while avoiding excessive light in the clear-cut.

## Materials and Methods

### Experimental Site

The experiment was conducted on the north end of St. Simon’s Island, Georgia at Cannon’s Point Preserve (N 31°15’29“ W 81°20’45”), which is a 246-ha wilderness tract with approximately 50 ha dominated by abandoned pine plantations (mostly *P. taeda* L. with some *P. elliotti* Englem.). Tree rings and cores indicated that the pine stands were approximately 50 years old. In 2015 and 2016, areas of natural and planted pines affected by southern pine beetles were clear-cut to salvage timber and reduce the southern pine beetle outbreak.

Soils at Cannon’s Point Preserve are a mixture of fine sandy soils dominated by Mandarin fine sand and Cainhoy fine sand, 0–5% slopes. Pottsburg sand and Rutledge fine sand are also present (NRCS, 2017). At each plot, four soil samples were composited to evaluate physical and chemical characteristics using Mehlich III extraction (Brookside Laboratories, New Brennan, Ohio). Soil characteristics were similar with slight differences creating variability across replicate blocks (Table 1).

**Table 1:** Mean (± SE) of initial soil parameters using Mehlich III extraction.

Chemical characteristic	
Organic matter (%)	2.1 (± 0.3)
pH	4.6 (± 0.3)
CEC (ME 100g^−1^)	2.2 (± 0.7)
Estimated nitrogen (kg ha^−1^)	67.8 (± 5.1)
Soluble sulfur (ppm)	8.5 (± 1.0)
Phosphorous (ppm)	99.6 (± 24.5)
Potassium (ppm)	17.3 (± 1.4)
Magnesium (ppm)	35.8 (± 4.6)
Calcium (ppm)	257.1 (± 93.3)
Sodium (ppm)	35.7 (± 1.8)

This region receives an average annual precipitation of 114 cm and average annual temperature was 20.0°C. During the study period 2017–2018, average annual precipitation was 90 cm and temperature was 21.0°C (Sapelo Island National Estuarine Research Reserve Meterological Monitoring, 2018; U.S. Climate Data, 2018). Hurricane Irma (September 2017; 7 months into experiment) resulted in temporarily increased precipitation, saltwater inundation, salt spray, and strong winds across the region, yet no damage to our experimental site.

### Experimental Design and Treatments

A randomized complete block design with a split-plot structure was used for this study. The whole plot factor consisted of four overstory densities (clear-cut, heavy thin, light thin, and non-thinned). The subplot factor was two levels of vegetation control (no vegetation removal and 2 years of vegetation removal). A total of 25 seedlings were planted within each subplot. All treatment combinations were replicated by four blocks resulting in 800 total seedlings.

Overstory density treatments were randomly applied to a 66 × 44 m area. Within the treated area, 26 × 14 m research plots were established. All plots were fenced (2.5 m height) to exclude white-tailed deer (*Odocoileus virginianus* Zimm.) as herbivory is cited as a limiting site factor in maritime forest restoration (Thyroff et al., 2019). Overstory density treatments were installed by modifying the basal area of the original pine overstory. Target basal areas were clear-cut at 0 m^2^ ha^−1^, heavy thin at 4–9 m^2^ ha^−1^, light thin at 18–23 m^2^ ha^−1^, and non-thinned at 27+ m^2^ ha^−1^. Logging operations to implement overstory treatments were completed in December 2016. Target basal areas were monitored by a contracted forester and logger. Additionally, all mid-story trees, understory vegetation, and large slash were removed immediately after the harvest activity to reduce possible confounding effects. Subplots requiring vegetation control over 2 years were done so throughout both growing seasons using mechanical methods (i.e., brush saws and hand clippers).

### Plant Material

One-year-old *Q. virginiana* bare-root seedlings were planted in February 2017. Seedlings were obtained from Superior Trees in Lee, Florida with a Louisiana seed source. From baseline morphology analysis (*n* = 20), mean seeding diameter was 5 mm (± 0.20), mean seedling height was 54 cm (± 2.00), and root to shoot dry mass (g) ratio was 0.89 (± 0.76). Seedlings were sorted prior to planting and randomly assigned treatments. Seedlings were hand planted with planting bars at 2-m spacing. To maintain planting density and interspecific seedling competition, a perimeter of buffer trees was planted 2 m from the research seedlings.

### Plot Characteristics

For each plot, basal area and canopy closure data were collected in summer 2017. All mature tree species within each fenced plot and the buffer areas around them were identified and measured for diameter at breast height (DBH; 1.37 m) to the nearest centimeter. DBH of mature trees were used to calculate the basal area. Three hemispherical photographs were taken under homogeneous diffuse sky conditions and across the centerline, working from west to east cardinal directions, at approximately 4.7-m intervals (1/3 of the plot width). Photographs were analyzed with CIMES software (Gonsamo et al., 2011) to determine percent canopy closure.

To record soil moisture and temperature, each plot had an Em50 digital data logger with two 5TM sensors (Decagon, Pullman, Washington) located in the subplot center. Each sensor was installed at a depth of 25 cm and recorded measurements every 2 h. Four of the plots (all of block 1) had additional sensors installed; photosynthetically active radiation (PAR) sensors to monitor light and VP4 sensors to capture air temperature and relative humidity recorded every 2 h. At the peak of vegetation cover on site (September 2018), five seedlings from each treatment (160 total) were randomly selected for a 1-m^2^ plot vegetation survey to assess percent competing vegetation cover, mean height of competition, and top competing species within each plot.

### Seedling Performance

At the time of planting (February 2017), measurements of ground line diameter and height to last live bud were recorded. At the end of the growing seasons (November 2017 and 2018), survival, diameter, height, and crown width were recorded. Survival was recorded as a binary response; “alive” included seedlings with any number of green leaves. At the end of the second growing season (November 2018), foliar nitrogen (N) was determined by randomly sampling seven seedlings per subplot, resulting in 224 total sampling units. Three leaves per seedling were collected and composited, dried at 60°C for 72 h, weighed, pulverized in vials with stainless steel balls, and analyzed with an ECS 4010 CHNSO Analyzer (Costech, Valencia, California).

### Ecophysiology and Leaf Trait Measurements

Gas exchange, specific leaf area (SLA), stomatal density, stomatal pore index (SPI), and maximum theoretical stomatal conductance (g_max_) were measured during the growing season (June 2018). Leaf gas-exchange was measured with a portable LI-6400XT (LI-COR Biosciences, Lincoln, Nebraska) to create light response curves. Two *Q. virginiana* seedlings were randomly selected per subplot, resulting in 64 total sampling units. One upper-canopy, fully expanded, recently mature leaf per tree was measured between the hours 10:00 and 14:00. Light levels used to create light response curves were: 1,600, 1,400, 1,200, 1,000, 800, 600, 400, 300, 200, 100, 50, 0 (µmol m^−2^ s^−1^). Infrared gas analyzers of the LI-6400XT (IRGAs; reference and sample) were matched at the beginning and end of each light curve measurement. Relative humidity (∼ 60%), vapor pressure deficit (<3.0 kPa), and temperature (leaf and block) were monitored for consistency. The gas exchange data point was taken after sample gas values (H_2_O and CO_2_) and net CO_2_ assimilation were stable, based on coefficient of variation. *Q. virginiana* leaves did not fully fill the 2 × 3 cm LI-6400XT leaf chamber, therefore, gas-exchange measurements were adjusted for actual leaf areas. Leaf areas were determined from a photo of the leaf in the chamber using ImageJ (National Institutes of Health, Bethesda, Maryland). Light response curves were created by plotting net CO_2_ assimilation (A_N_, µmol CO_2_ m^−2^ s^−1^) against PAR. The curves were fitted to a non-rectangular hyperbola (SigmaPlot V11.0, Systat Software, San Jose, California). Methodology to calculate final parameters from the model followed Chartier and Prioul (1976). Final parameters were used to calculate light compensation (µmol m^−2^ s^−1^) and light saturation (µmol m^−2^ s^−1^) points.

SLA, stomatal density, SPI, and g_max_ were sampled from the same four selected *Q. virginiana* seedlings per plot used for gas-exchange measurements. Three selected, upper-canopy, fully expanded, recently mature leaves were used for each seedling. In the non-thinned overstory some seedlings did not have many leaves, therefore in those cases only two leaves were collected. SLA was calculated by dividing leaf area by leaf mass (cm^2^ g^−1^). Collected whole leaves were scanned to measure leaf area (cm^2^) using ImageJ. Leaves were dried at 60°C for 48 h then weighed for leaf mass (g).

Impressions of the abaxial leaf surface were made in the middle of each leaf, midway between the midrib and the leaf margin. Leaf impressions were made on microscope slides using cyanoacrylate. Five leaf impression images (DCM 900 microscope CMOS Camera, Oplenic Optronics, Hangzhou, China) were taken of a 0.19 × 0.14 mm (0.0266 mm^2^) area under 40× magnification using a microscope (BH-2 microscope, Olympus, Tokyo, Japan). Stomatal counts were conducted using ImageJ and the cell counter plug-in (Kurt De Vos, University of Sheffield). For unbiased counting, all whole stomata were counted within the impression image area and stomata partially within the image were only counted on the top and right sides of the image area. Stomatal density (mm^−2^) was calculated by dividing the number of stomata in the image by image area. To calculate SPI and g_max_, stomatal lengths and widths were calculated from the leaf impressions using ImageJ and using the Feret’s diameter measurement (**Supplementary Appendix A**). SPI was calculated by multiplying stomatal density by stomatal length squared, while g_max_ (mmol m^−2^ s^−1^) was calculated using stomatal length and width following equations from McElwain et al. (2016) methodology.

### Statistical Analyses

All data was analyzed with R software version 3.5.3 (R Core Team, 2019) using: lme4 package (Bates et al., 2015) for general linear models, linear regressions, and logistic regression; nlme (Pinheiro et al., 2018) package for repeated measures models; multcomp package (Hothorn et al., 2008) for pairwise comparisons. Plot characteristics (basal area, canopy closure, and data loggers) were analyzed with general linear mixed models, with overstory as the fixed factor and block as a random factor. A logistic regression model was used to analyze survival with overstory, vegetation control, and interaction as fixed factors and block as a random factor. Diameter, height, and crown width were analyzed with repeated measures general linear mixed models with overstory, vegetation control, time, and resulting interactions as fixed factors; block and individual tree as random factors. Foliar N, light response curves including light compensation/saturation points, leaf traits (i.e., SLA, stomatal density, SPI, g_max_), and vegetation survey dependent variables were analyzed separately with general linear mixed models, with overstory, vegetation control, and interaction as fixed factors and block as a random factor. Linear regression models were used to analyze light saturation points and growth parameters. Residuals from all response variables were tested to ensure normality and homogeneity of variance. Crown width did not meet assumptions and data was square root transformed. For all analyses, when significant treatment effects were detected (p ≤ 0.05), Tukey’s HSD test was used to test for pairwise comparisons (α = 0.05). All statistical output results are provided in **Appendices B** and **C**. Although the number of sampling units from each experimental unit varied across measurements (per details above), the number of experimental units was always n = 4.

## Results

### Plot Characteristics

**Figure 1:**
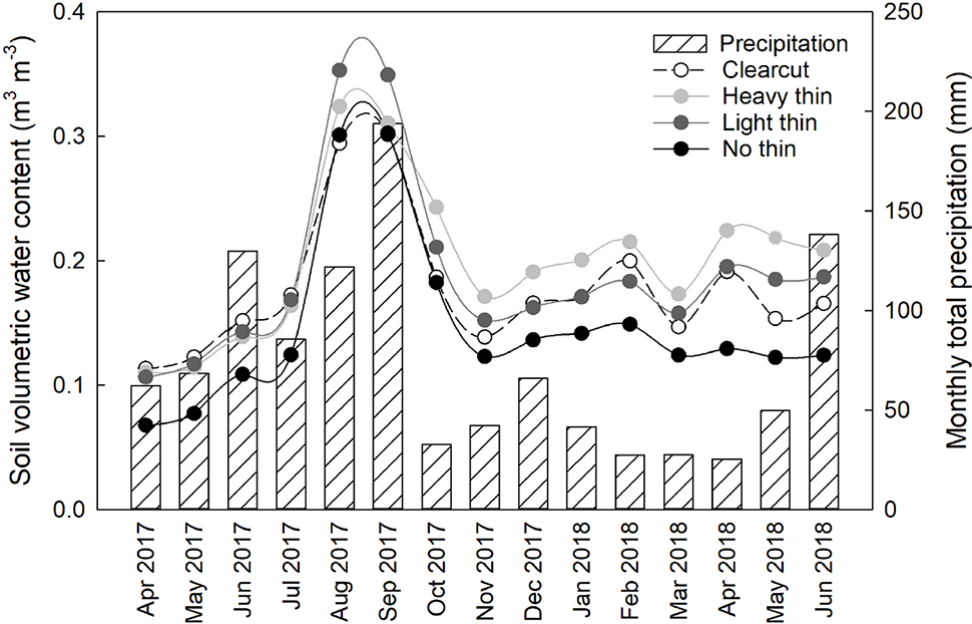
Mean soil volumetric water content (m^3^ m^−3^) from all plots, averaged by overstory treatment from April 2017 to June 2018. Volumetric water content was recorded every 2 h. Secondary y axis, monthly total precipitation (mm) from Sapelo Island National Estuarine Research Reserve Meteorological Monitoring station from April 2017 to June 2018.

Basal area (m^2^ ha^−1^), canopy closure (%), light (PAR), mean air temperature (°C), and mean soil temperature (°C) followed a progression of overstory density (Table 2). Clear-cuts had the lowest basal area and canopy closure, resulting in greatest PAR. This pattern was consistent along the light progression with thinned plots having intermediate basal area and canopy closure. Non-thinned plots had the greatest basal area and canopy closure, resulting in the lowest PAR. Mean air and soil temperature increased with increased thinning density resulting in highest temperatures in clear-cut plots. From April 2017 to May 2018, average soil moisture was consistently greater in vegetation control subplots than non-vegetation control subplots. Soil moisture peaked in late summer/early autumn and was often greatest in heavy thin plots, followed by clear-cut and light thin, and lastly non-thinned plots (**Figure 1**).

**Table 2:** Mean (± SE) target basal area from logging operation, basal area from stand inventory, canopy closure from hemispherical photos.

	Basal area (m^2^ ha^−1^)	Canopy closure (%)	PAR (μmol m^−2^ s^−1^)	Air temperature (°C)	Soil temperature (°C)
Clear-cut	0 (± 0.0) a	0 (± 0.00) a	803 (± 9.3) a	25.5 (± 0.07) a	23.8 (+ 1.00) a
Heavy thin	16.9 (± 2.3) b	60 (± 0.05) b	446 (± 4.4) b	22.4 (± 0.09) b	22.1 (+ 0.45) ab
Light thin	24.2 (± 1.5) b	67 (± 0.04) b	278 (± 7.5) b	20.1 (± 0.11) c	21.5 (+ 0.20) b
No thin	33.9 (± 3.5) c	78 (± 0.05) b	144 (± 2.5) c	20.7 (± 0.10) c	21.4 (+ 0.40) b

Mean (± SE) PAR levels, air temperature, and soil temperature from data loggers. Different letters indicate significant differences among treatments (α = 0.05).

**Figure 2:**
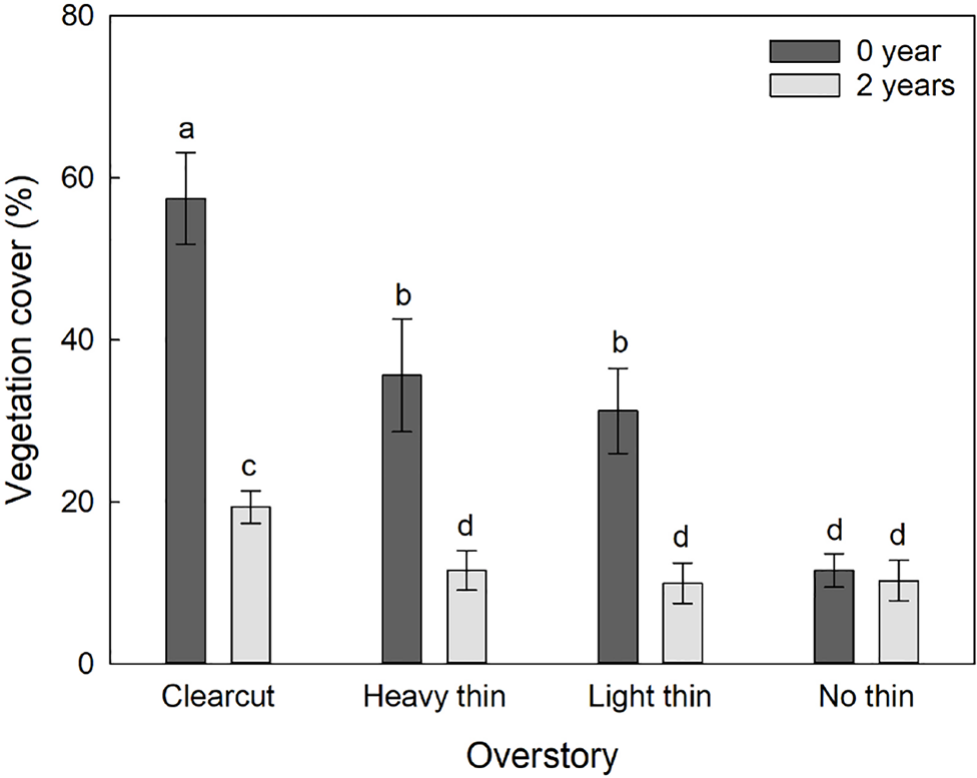
Mean (± SE) vegetation cover (%) of competing vegetation in a 1-m^2^ survey around *Quercus virginiana* seedlings planted in clear-cut, heavy thin, light thin, no thin plots taken at the end of the second growing season. Competing vegetation removed for 0 or 2 years. Different letters indicate significant differences among treatments (α = 0.05).

The interaction of overstory and vegetation control was significant for percent vegetation cover (F_3,54_ = 6.84, p = 0.001) as the effect of vegetation control was different between the overstory treatments. Vegetation control decreased percent vegetation cover in clear-cut, heavy thin, and light thin plots, but had no effect in non-thinned plots (**Figure 2**). Additionally, height of competing vegetation was 58.3 cm (± 14.4) in non-vegetation control subplots compared to 18.8 cm (± 3.0) in vegetation control subplots (F_1,57_ = 35.56, p < 0.001). Top competing species included: *Ilex vomitoria*, *Paspalum notatum*, *Rubus trivialis*, *Vitis rotundifolia*, and *Morella cerifera*.

### Seedling Performance

Initial height and diameter of planted seedlings were similar across all treatments with an average height of 48 cm (± 0.9) and an average diameter of 3.8 mm (± 0.1). Overall survival after two growing seasons was 75.5% (± 1.9). The treatment interaction was not significant, however the main effect of overstory was significant with increased survival with increased thinning intensity (X^2^_3,794_ = 9.86, p = 0.020); clear-cut and heavy thin plots had the highest survival at 81.5 and 81.0%, light thin was intermediate at 73.0%, and non-thinned had the lowest survival at 65.5%.

**Figure 3:**
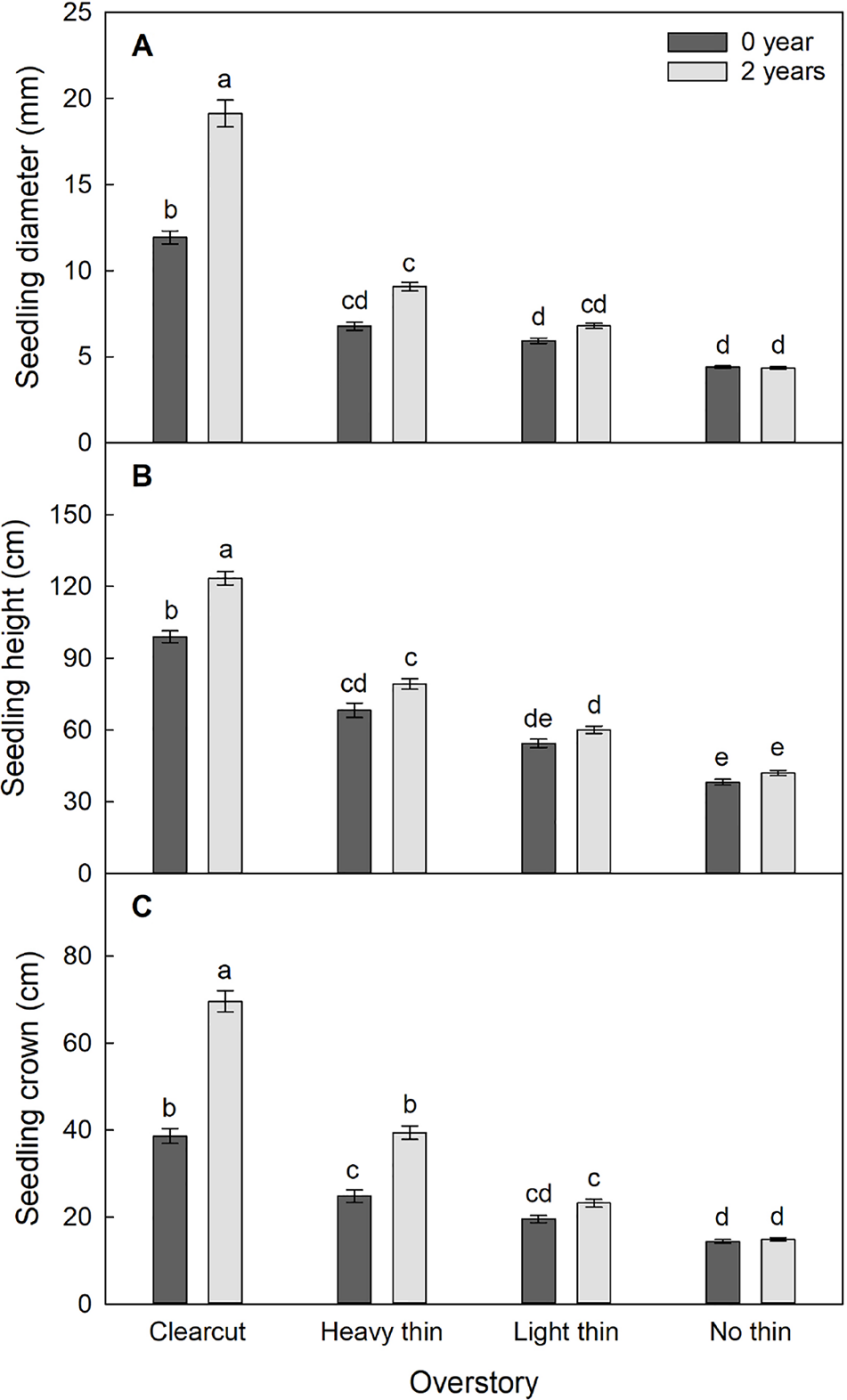
Mean (± SE) **(A)** diameter (mm), **(B)** height (cm), and **(C)** crown width (cm) growth of *Quercus virginiana* seedlings after the second (2018) growing season. Planted in overstory density (clear-cut, heavy thin, light thin, or no thin plots) and vegetation control treatments (0 or 2 years of subplots). Different letters indicate significant differences among treatments (α = 0.05).

The interaction of overstory, vegetation control, and time was significant for diameter (F_6,595_ = 13.30, p < 0.001), height (F_6,1217_ = 3.22, p = 0.004), and crown width (F_3,587_ = 13.00, p < 0.001). The effect of vegetation control differed among the overstory density treatments and the effects varied over time. Overall diameter, height, and crown width increased with increased thinning intensity. Vegetation control was most beneficial for seedlings in clear-cut plots followed by heavy thin plots and had no effect in non-thinned plots (**Figure 3**).

Seedling diameter increased by 400% in clear-cut plots with vegetation control and 200% in clear-cut plots without vegetation control, while marginal growth occurred in non-thinned plots regardless of vegetation control treatment. Seedling height and crown width increased by 200% in clear-cut plots with vegetation control and 100% in clear-cut plots without vegetation control, while non-thinned plots had little growth regardless of treatment. Dieback occurred frequently in the non-thinned plots, which resulted in negative relative heights after two growing seasons.

### Ecophysiology and Leaf Trait Measurements

**Figure 4:**
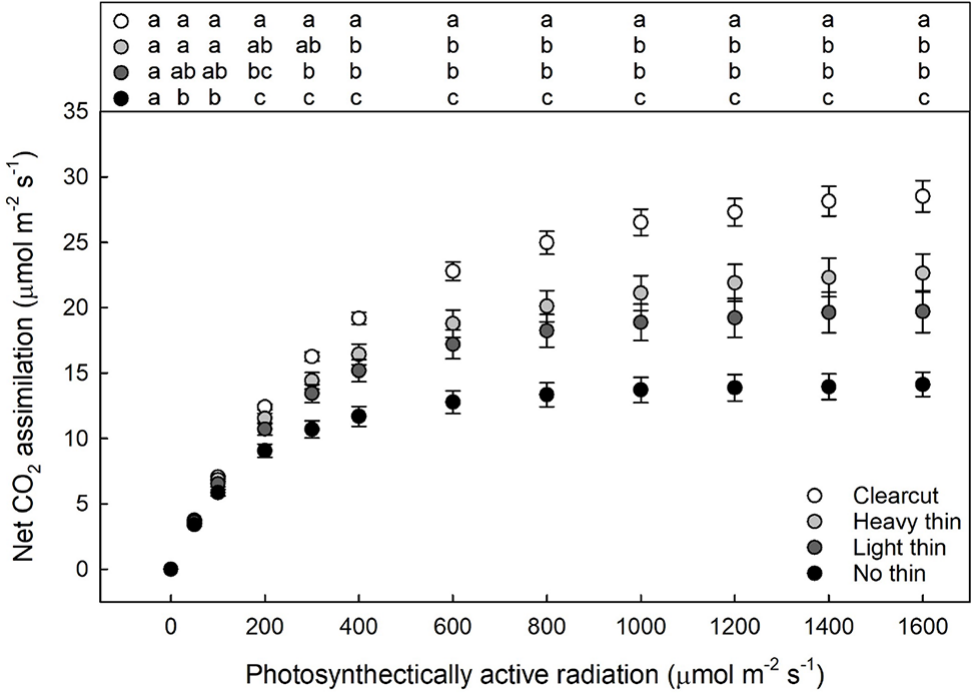
Mean light response curves (net photosynthesis plotted by photosynthetically active radiation) of *Quercus virginiana* seedlings planted either in clear-cut, heavy thin, light thin, or no thin plots taken in June 2018. Different letters indicate significant differences among treatments (α = 0.05).

For foliar N, only main effects were significant showing increased foliar N with increased thinning intensity (F_3,217_ = 24.06, p < 0.001) and increased foliar N with vegetation control (F_1,217_ = 34.94, p < 0.001). While the interaction was non-significant the overstory main effect was significant for the calculated light compensation (F_3,57_ = 8.10, p < 0.001) and light saturation points (F_3,57_ = 23.56, p < 0.001). With increased thinning intensity, light compensation and saturation points increased. Net CO_2_ assimilation was greatest in clear-cut plots, intermediate in heavy and light thin, lowest in non-thinned plots (**Figure 4**), and positively related to growth parameters (**Supplementary Appendix D**). Additionally, the vegetation control main effect was significant only for light saturation point with greater light saturation points in subplots with vegetation control compared to subplots without vegetation control (F_1,57_ = 5.56, p = 0.022).

**Figure 5:**
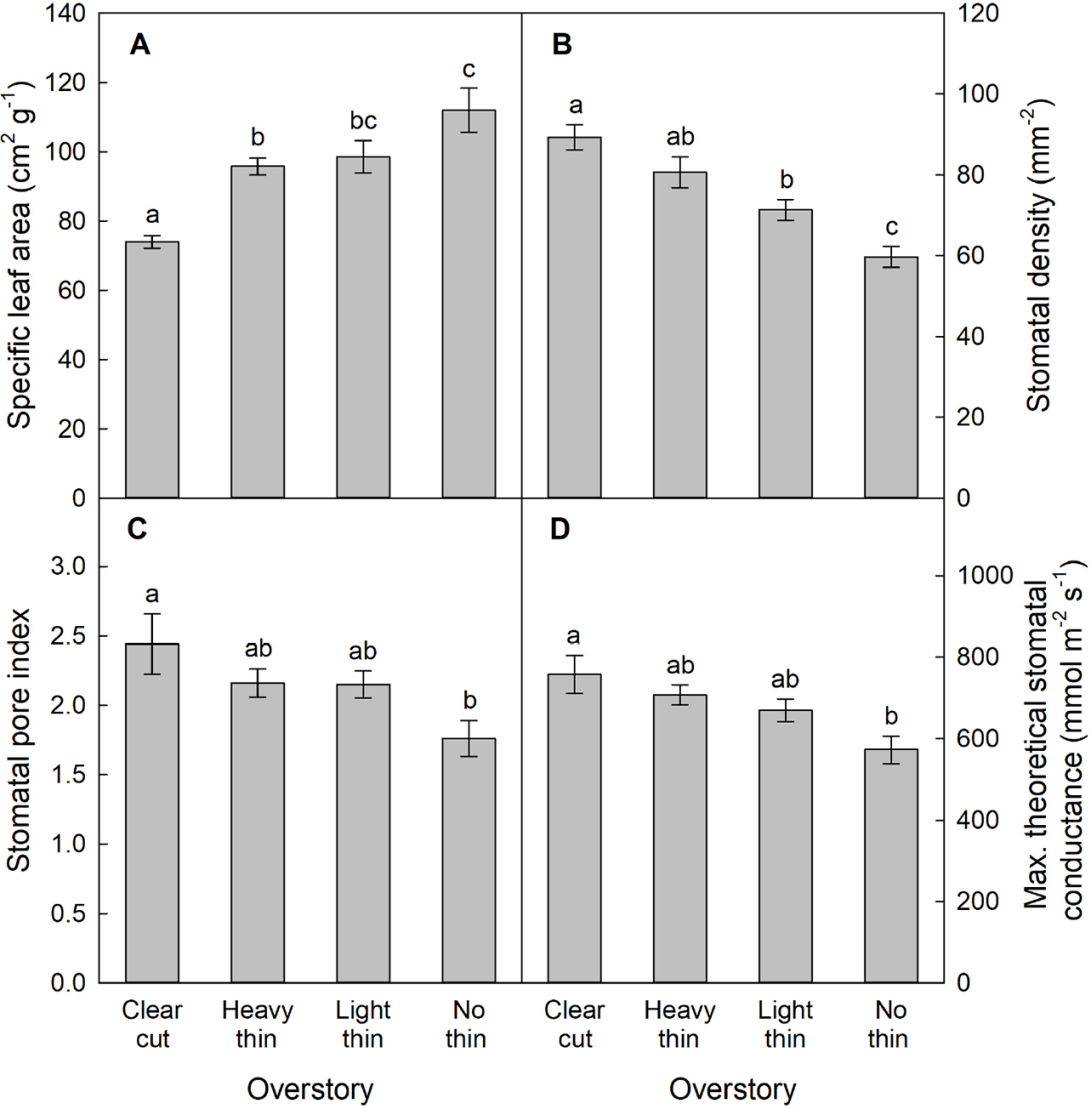
Mean (± SE) **(A)** specific leaf area (cm^2^ g^−1^), **(B)** stomatal density (stomata mm^−2^), **(C)** stomatal pore index, and **(D)** maximum theoretical stomatal conductance (g_max_, mmol m^−2^ s^−1^) of *Quercus virginiana* seedlings planted in clear-cut, heavy thin, light thin, or no thin plots. Different letters indicate significant differences among treatments (α = 0.05).

**Figure 6:**
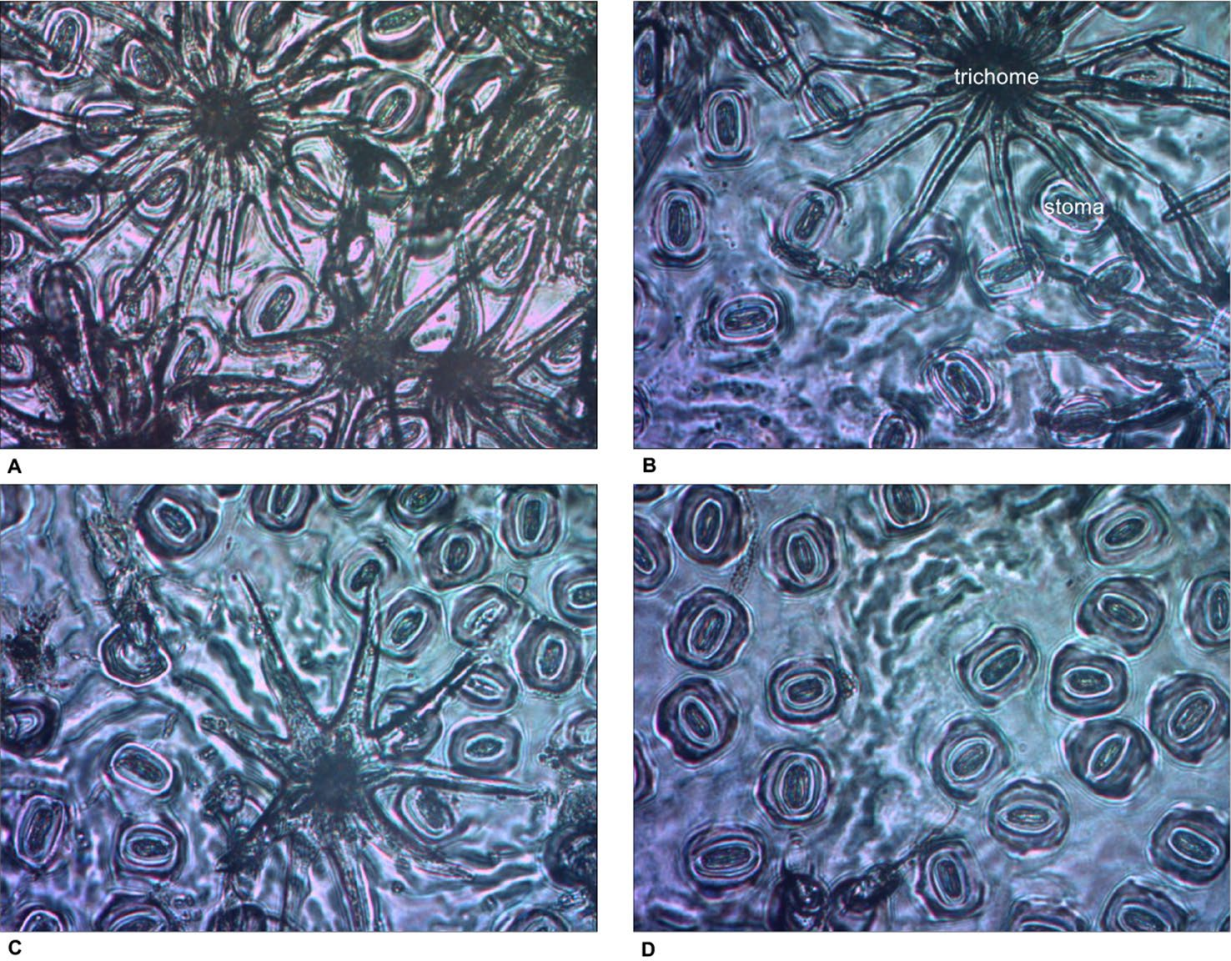
Impression images of abaxial trichomes *Quercus virginiana* seedlings planted in each of the overstory treatments [**(A)** clear-cut, **(B)** heavy thin, **(C)** light thin, and **(D)** no thin].

For SLA, stomatal density, SPI, and g_max_, only main effects were significant. With increased thinning intensity, SLA decreased (F_3,57_ = 12.60, p < 0.001), while stomatal density (F_3,57_ = 21.20, p < 0.001), SPI (F_3,57_ = 24.19, p < 0.001), and g_max_ (F_3,57_ = 24.48, p < 0.001) all increased (**Figure 5**). Vegetation control resulted in decreased SLA (F_1,57_ = 6.28, p = 0.015), and increased stomatal density (F_1,57_ = 5.37, p = 0.024), SPI (F_1,57_ = 6.55, p = 0.013), and g_max_ (F_1,57_ = 5.69, p = 0.020). More abaxial trichomes (i.e., leaf hairs) occurred on stomatal density impression images from clear-cut plots followed by heavy thin, light thin, and lastly no trichomes in non-thinned plots (F_3,57_ = 6.38, p < 0.001; **Figure 6**).

## Discussion

### Light and Competing Vegetation

Removal of stand basal area affects environmental characteristics such as light, temperature, and soil moisture, which influence seedling performance (Gil-Pelegrín et al., 2017b; Villar-Salvador, 2016). In our study, the pattern reflected in the PAR results (clear-cut had greatest PAR, followed by the thinned overstories, and lastly non-thinned) corresponded with increased seedling survival, diameter, height, crown width, and foliar N. Growth parameters were consistently greatest in clear-cut plots, intermediate in thinned plots, and lowest in non-thinned plots, rather than peaking in the thinned treatments as we hypothesized. Foliar N, which is an essential macronutrient for seedling establishment and performance (Abrams and Mostoller, 1995; Soto et al., 2017), was significantly greater in leaves of seedlings in clear-cut plots followed by the thinned overstories, indicating greater accessibility of this limiting resource to seedlings (Kobe, 2006; Uscola et al., 2015). Similar to other studies, seedlings in less dense overstory treatments were able to use available resources, and thereby increase photosynthesis and growth (Gómez-Aparicio et al., 2006; Cooper et al., 2014; Soto et al., 2017). Supporting our results, *Q. virginiana* has been reported as a fast growing species that preferentially allocates resources to aboveground biomass development (Cavender-Bares and Pahlich, 2009).

In a dense overstory, such as the non-thinned plots, soil moisture and nutrients (e.g., N as seen with low foliar N concentrations) are typically limited because of canopy tree dominance and competition (Cooper et al., 2014). Seedlings in non-thinned plots with dense overstories may have arrested development due to low light levels preventing their progression from seedlings to saplings (Zavala et al., 2011; Soto et al., 2019). Artificially regenerated *Q. virginiana* did not appear to benefit from the shade of the pine plantations as identified with *Q. ilex* L. (Gómez-Aparicio et al., 2009); however, this advantage may be expressed during a prolonged drought or on drier coastal sites or if studying natural regeneration. Thus, effects may also be due to the density of the pine plantations (33.9 m^2^ ha^−1^ basal area) in our study. Sites with pine overstory densities closer to the heavy thin treatment (16.9 m^2^ ha^−1^ basal area) may see the benefit of directly underplanting in a plantation.

As hypothesized, we found a consistent interaction between overstory and vegetation control treatments, with vegetation control benefiting growth parameters in clear-cut plots and not in non-thinned plots (**Figure 3**). Without thinning, light and soil moisture were more limiting to *Q. virginiana* performance than understory vegetation competition. Semi-evergreen *Quercus* spp. have varying photosynthetic responses to different environments, with a range of growth rates and shade tolerance (Gratani et al., 2003; Cavender-Bares and Ramírez-Valiente, 2017; Villar et al., 2017). Within the clear-cut and heavy thinning treatments, higher PAR contributed to understory vegetation becoming more abundant and thus more competitive (Ter-Mikaelian et al., 1999). In our study, vegetation control promoted seedling growth, particularly on sites with more light (i.e., clear-cut and heavy thin). The significant interaction illustrated a shift in pressure from light limited environments to resource limited environments (i.e., greater foliar N concentrations and increased soil moisture in vegetation control subplots). Overall, our results align with past studies indicating that vegetation control enhances seedling establishment and performance, particularly when released from dense overstories (Wagner and Zasada, 1991; Fleming et al., 2006). In cases where competing vegetation is expected to be high and vegetation control is not feasible, however, maintaining an overstory may reduce competition and the need for costly and sometimes controversial (i.e., herbicide) competing vegetation removal (Thiffault and Roy, 2011; Löf et al., 2014; Villar-Salvador, 2016).

### Ecophysiology and Leaf Trait Responses

Trees are long lived organisms that may experience several environmental changes and associated stresses and plasticity is an adaptive strategies to promote survival under environmental fluctuations (Cavender-Bares and Ramírez-Valiente, 2017; Gil-Pelegrín et al., 2017a). Our results indicate that *Q. virginiana* can acclimate to varying environments as maximum net CO_2_ assimilation occurred in clear-cuts (**Figure 4**), which aligns with the increased growth and foliar N in clear-cut plots. With higher light compensation and saturation points, seedlings in clear-cuts took longer to achieve positive CO_2_ assimilation, but were able to utilize increased PAR with higher net CO_2_ assimilation rates. Similar to studies with other oaks, *Q. virginiana* seedlings responded to the other overstory treatments in a linear progression with respect to light availability (Cavender-Bares and Pahlich, 2009; Cooper et al., 2014). Seedlings in the non-thinned plots reached positive net CO_2_ assimilation quickest at the lowest PAR; however, non-thinned seedlings also reached light saturation point quicker at a lower PAR. Net CO_2_ assimilation, therefore, was limited and lowest for seedlings in non-thinned plots. Greater net CO_2_ assimilation rates were supported by more stomata, not larger stomata (**Supplementary Appendix A**), resulting in greater gas exchange potential as seen with stomatal density, SPI, and g_max_. Ability to regulate stomatal development (stomatal density and SPI) and capacity for high g_max_ rates leads to seedling acclimation to a wide range of environments. Additionally, seedlings in more shaded environments with decreased SPI can allow for higher water use efficiency (Yoo et al., 2009; McElwain et al., 2016; Liu et al., 2017). In other studies, Mediterranean evergreen oaks such as *Quercus oleoides* Schltdl. & Cham. and *Quercus ilex* have also shown phenotypic plasticity in growth rates, CO_2_ assimilation, and leaf development (Cavender-Bares and Ramírez-Valiente, 2017; Gil-Pelegrín et al., 2017b; Ramírez-Valiente et al., 2017). Niinemets and Valladares (2006) classified *Q. virginiana* as drought-tolerant and intermediate in shade tolerance. Not only is maximizing performance under high-light conditions beneficial, but also the capability of shade adaptation is beneficial to seedling survival (Valladares and Niinemets, 2008). Similarly, *Quercus velutina* Lam. is a drought-tolerant and light demanding oak for which Ashton and Berlyn (1994) showed great leaf anatomical plasticity with high net CO_2_ assimilation compared to other temperate deciduous oaks.

Leaf variation of *Quercus* spp. tends to be on the lower end of the leaf economic spectrum, aligning with early to mid-successional classification (Wright et al., 2004; Niinemets and Valladres, 2006; Gil-Pelegrín et al., 2017b). Following a conservative resource strategy, lower SLA leaves on semi-evergreen oaks can help to maintain function and extend photosynthesis (Cavender-Bares and Ramírez-Valiente, 2017). Thicker leaves are also more resistant to environmental stressors such as aridity, freezing temperatures, and solar radiation (Cavender-Bares and Ramírez-Valiente, 2017; Gil-Pelegrín et al., 2017b; Pemán et al., 2017). Whereas SLA was negatively associated with CO_2_ assimilation, stomatal density was positively associated with CO_2_ assimilation. Similarly, Ramírez-Valiente et al. (2017) found that SLA was negatively associated with net CO_2_ assimilation and *Q*. seedling growth. In clear-cut plots with low SLA (i.e., smaller, thicker leaves), seedling growth was greatest. Higher stomatal density and SPI increases gas exchange potential and g_max_, which increases net CO_2_ assimilation (Wright et al., 2004; McElwain et al., 2016). Along with increased gas exchange potential, comes increased risk of desiccation; therefore, a trade-off is necessary to maximize performance, which was seen in the opposite associations of SLA and stomatal density with net CO_2_ assimilation. Trichomes are commonly found in many *Quercus* spp. and trichomes in the abaxial surface may be a mechanism to reduce water loss (Bickford, 2016; Gil-Pelegrín et al., 2017b) as seen in the higher light plots (**Figure 6**). Additionally, a smaller leaf typically has a thinner leaf boundary layer, which facilitates cooling in drier and warmer climates (e.g., clear-cuts) (Gil-Pelegrín et al., 2017b). In our experiment, *Q. virginiana* showed phenotypic plasticity for growth rates, gas exchange, and leaf development in response to silvicultural treatments that modified seedling environments as was observed in similar evergreen oak regeneration studies (Ramírez-Valiente et al., 2015).

## Conclusions

We demonstrated that *Q. virginiana* is capable of acclimating to varying resource availability, with a high tolerance to full sunlight. Developmental responses of underplanted *Q. virginiana* did not follow the predicted trend of peaking in the heavy/light thin pine plantation overstory, but rather in clear-cuts. Nonetheless, thinning is among the most effective practices for preventing or mitigating pine beetle outbreaks by creating barriers to population growth and spread (Nowak et al., 2015; Asaro et al., 2017). Additionally, a canopy buffering-effect in thinned stands may be more beneficial for oaks on drier sites or during prolonged drought (García-Plazaola et al., 2017). Thus, rather than prescribing a single treatment (e.g., clear-cut) that optimizes *Q. virginiana* development, an alternative may be to prescribe several treatments that result in cost-effective restoration and reduce the need for costly competing vegetation control. Forest species with high plasticity and rapid growth, such as *Q. virginiana* in our study, will obtain rapid canopy closure, allowing favorable conditions for establishment of associated mid-and late-seral maritime forest species (Gómez-Aparicio et al., 2009; Bertacchi et al., 2016), helping to create resilient, diverse, and complex forests (Löf et al., 2019).

## Data Availability Statement

The datasets generated for this study are available on request to the corresponding author.

## Author Contributions

ET designed and installed the experiment, collected and analyzed the data, and co-wrote the paper. OB helped to design and install the experiment, contributed to data analysis and interpretation, and co-wrote the paper. MM assisted with stomatal data collection and interpretation, and co-wrote the paper. DJ supervised the research, helped to design the experiment, and co-wrote the paper.

## Funding

Funding support was provided by the USDA National Institute of Food and Agriculture, McIntire Stennis projects IND011535 and NMSU1002447, Hardwood Tree Improvement and Regeneration Center, Fred M. van Eck Forest Foundation, John T. Harrington Forestry Center, and St. Simon’s Land Trust.

## Conflict of Interest

The authors declare that the research was conducted in the absence of any commercial or financial relationships that could be construed as a potential conflict of interest.
