# Supplementary material for: Unraveling Shade Tolerance and Plasticity of Semi-Evergreen Oaks: Insights From Maritime Forest Live Oak Restoration
Source: Front Plant Sci. 2019 Nov 20;10:1526. doi: 10.3389/fpls.2019.01526 (PMC6879670; doi:10.3389/fpls.2019.01526)

Appendix A. Mean (± SE) area, length, and width of *Q. virginiana* seedling stomata. Seedlings were planted in overstory density (clearcut, heavy thin, light thin, or no thin plots) and vegetation control treatments (0 or 2-yrs subplots). Non-significant differences among treatments (α = 0.05).

Overstory	Vegetation Control	Area (µm^2)	Length (µm)	Width (µm)
Clearcut	0 year	91.08 (± 15.91)	16.07 (± 1.04)	7.06 (± 0.79)
Heavy thin	0 year	90.88 (± 5.54)	15.69 (± 0.65)	7.35 (± 0.18)
Light thin	0 year	105.26 (± 5.75)	16.88 (± 0.76)	7.91 (± 0.09)
No thin	0 year	96.31 (± 3.49)	15.94 (± 0.43)	7.67 (± 0.12)
Clearcut	2 years	93.72 (± 11.89)	16.51 (± 0.93)	7.10 (± 0.59)
Heavy thin	2 years	89.19 (± 17.89)	16.10 (± 1.49)	6.84 (± 0.74)
Light thin	2 years	98.95 (± 7.73)	16.61 (± 0.70)	7.52 (± 0.33)
No thin	2 years	100.48 (± 6.20)	16.52 (± 0.51)	7.72 (± 0.30)

Appendix B. Repeated measures general linear mixed model results for diameter, height, crown width growth of *Q. virginiana* seedlings after the second (2018) growing season (Time/T). Planted in overstory density (Overstory/O) and vegetation control treatments (Veg. Ctrl./VC). Bolded p-values indicate significant differences (α = 0.05).

	Time	Overstory	Veg. Ctrl.	T × O	T × VC	O × VC	T × O × VC
Diameter	**<0.0001**	**<0.0001**	**<0.0001**	**<0.0001**	**<0.0001**	**<0.0001**	**<0.0001**
Height	**<0.0001**	**<0.0001**	**0.0005**	**<0.0001**	**<0.0001**	0.0560	**0.0038**
Crown width	**<0.0001**	**<0.0001**	**<0.0001**	**<0.0001**	**<0.0001**	**<0.0001**	**<0.0001**

Appendix C. General linear mixed model results for foliar nitrogen, light compensation, light saturation, specific leaf area, stomatal density, stomatal pore index, maximum theoretical stomatal conductance, vegetation cover, vegetation height. Planted in overstory density (Overstory/O) and vegetation control treatments (Veg. Ctrl./VC). Bolded p-values indicate significant differences (α = 0.05).

	Overstory	Veg. Ctrl.	O × VC
Foliar nitrogen	**<0.0001**	**<0.0001**	0.1582
Light compensation	**<0.0001**	0.8081	0.5039
Light saturation	**<0.0001**	**0.0219**	0.3473
Max. theoretical stomatal conductance	**<0.0001**	**0.0205**	0.4025
Specific leaf area	**<0.0001**	**0.0152**	0.3937
Stomatal density	**<0.0001**	**0.0241**	0.2943
Stomatal pore index	**<0.0001**	**0.0133**	0.4933
Vegetation cover	**<0.0001**	**<0.0001**	**0.0006**
Vegetation height	0.4021	**<0.0001**	0.6344

Appendix D. Linear regressions between light saturation points and seedling diameter (mm), height (cm), and crown width (cm) of *Q. virginiana* seedlings.

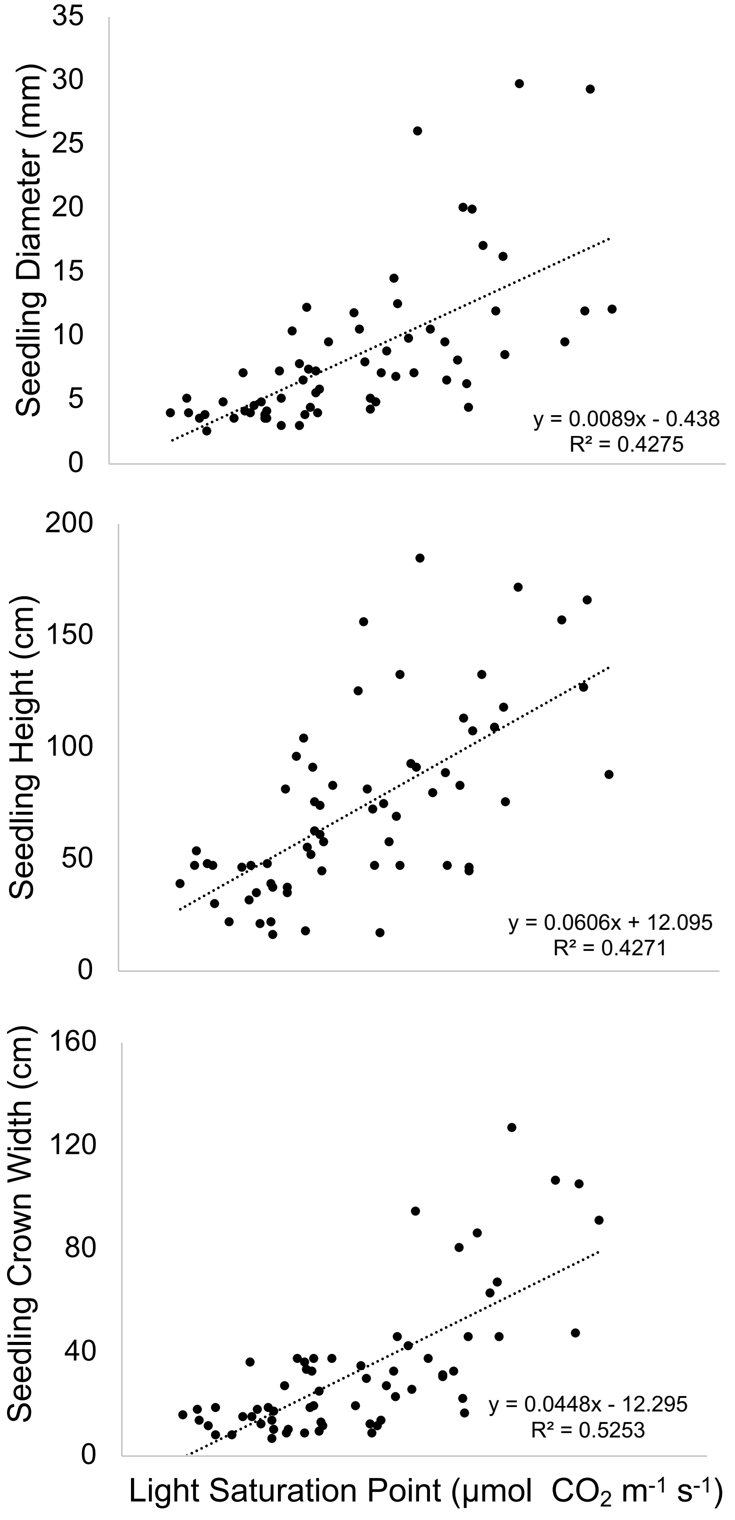

Supplement: Supplementary file 1 [file DataSheet_1.pdf]
